# The contribution of personalized video feedback to robotic partial nephrectomy training in realistic 3D tumor kidney models: design, production and implementation

**DOI:** 10.3389/fsurg.2025.1615817

**Published:** 2025-07-17

**Authors:** Ahmet Furkan Sarıkaya, Kayhan Tarım, Ersin Köseoğlu, Arif Özkan, İbrahim Can Aykanat, Baris Esen, Umut Can Karaarslan, Mustafa Müdüroğlu, Şevval Kanlı, Murat Can Kiremit, Yakup Kordan, Mevlana Derya Balbay, Tarık Esen, Serdar Aydın, Abdullah Erdem Canda

**Affiliations:** ^1^ Department of Urology, Koç University, Istanbul, Türkiye; ^2^ Department of Obstetrics and Gynecology, Koç University, Istanbul, Türkiye; ^3^ Research Center for Translational Medicine, Koç University, Sarıyer, Türkiye; ^4^ RMK AIMES, Rahmi M. Koc Academy of Interventional Medicine, Education, and Simulation, Istanbul, Türkiye

**Keywords:** robotic partial nephrectomy, 3D-printed kidney models, video feedback, surgical simulation training, urology resident education

## Abstract

**Background:**

Traditional surgical training relies on a master-apprentice model, but limitations such as restricted working hours and evolving surgical techniques have led to the integration of simulation-based training. Three-dimensional (3D) printing has emerged as a valuable tool for enhancing surgical education, offering patient-specific anatomical models that improve skill acquisition. Additionally, personalized video feedback may further refine training outcomes. This study investigates the impact of 3D-printed renal models and video-based feedback on skill acquisition in robotic partial nephrectomy training.

**Methods:**

Forty urology residents without prior robotic surgery experience participated in this study. After completing a standardized theoretical and simulation-based training program, they performed partial nephrectomy on 3D-printed kidney models. The participants were randomly assigned to two groups: one received personalized video feedback based on their recorded surgical performances, while the other proceeded without feedback. Their surgical performance was evaluated based on dissection time, renorrhaphy time, total console time, and the amount of healthy renal parenchyma removed. Statistical analysis was conducted to compare improvements between the groups and assess the impact of video feedback on skill development.

**Results:**

Initial comparisons between junior and senior residents showed no significant differences in their first operations, demonstrating that e-learning and proficiency-based simulation training effectively equalized skill levels before transitioning to realistic 3D model-based training. This suggests that structured preparatory training with objective performance targets can enhance learning outcomes in surgical simulation. Additionally, 3D-printed models provide a significantly more cost-effective alternative to cadaveric and animal-based training, making high-quality surgical education more accessible and scalable. Residents who received video feedback demonstrated a greater percentage improvement in dissection time compared to the control group (46.63% vs. 23.62%, *p* = 0.043). The amount of healthy renal parenchyma removed significantly decreased in the video feedback group (*p* = 0.048), indicating improved surgical precision. No significant differences were observed in renorrhaphy times between the two groups, suggesting that video feedback primarily enhanced dissection skills.

**Conclusion:**

The integration of 3D-printed anatomical models with personalized video feedback enhances skill acquisition in robotic partial nephrectomy training. Video feedback significantly improves surgical precision by reducing unnecessary parenchymal removal and accelerating dissection time. These findings support the use of patient-specific 3D models and targeted feedback as cost-effective and scalable strategies to optimize surgical education and shorten the learning curve for complex procedures.

## 1 Introduction

The traditional surgical education model has long been grounded in the master-apprentice relationship, yet modern educational approaches began emerging in the late 19th century. This model, largely based on the progressive responsibility system initiated by William Stewart Halsted at Johns Hopkins Hospital, has been a cornerstone in training surgeons for decades (1, 2). Nevertheless, evolving challenges have necessitated the development of new approaches in surgical education. Legal limitations on weekly working hours, an increasing bureaucratic burden, and the diversification of surgical techniques and equipment have all contributed to diminishing the effectiveness of traditional training methods.

**Figure 1:**
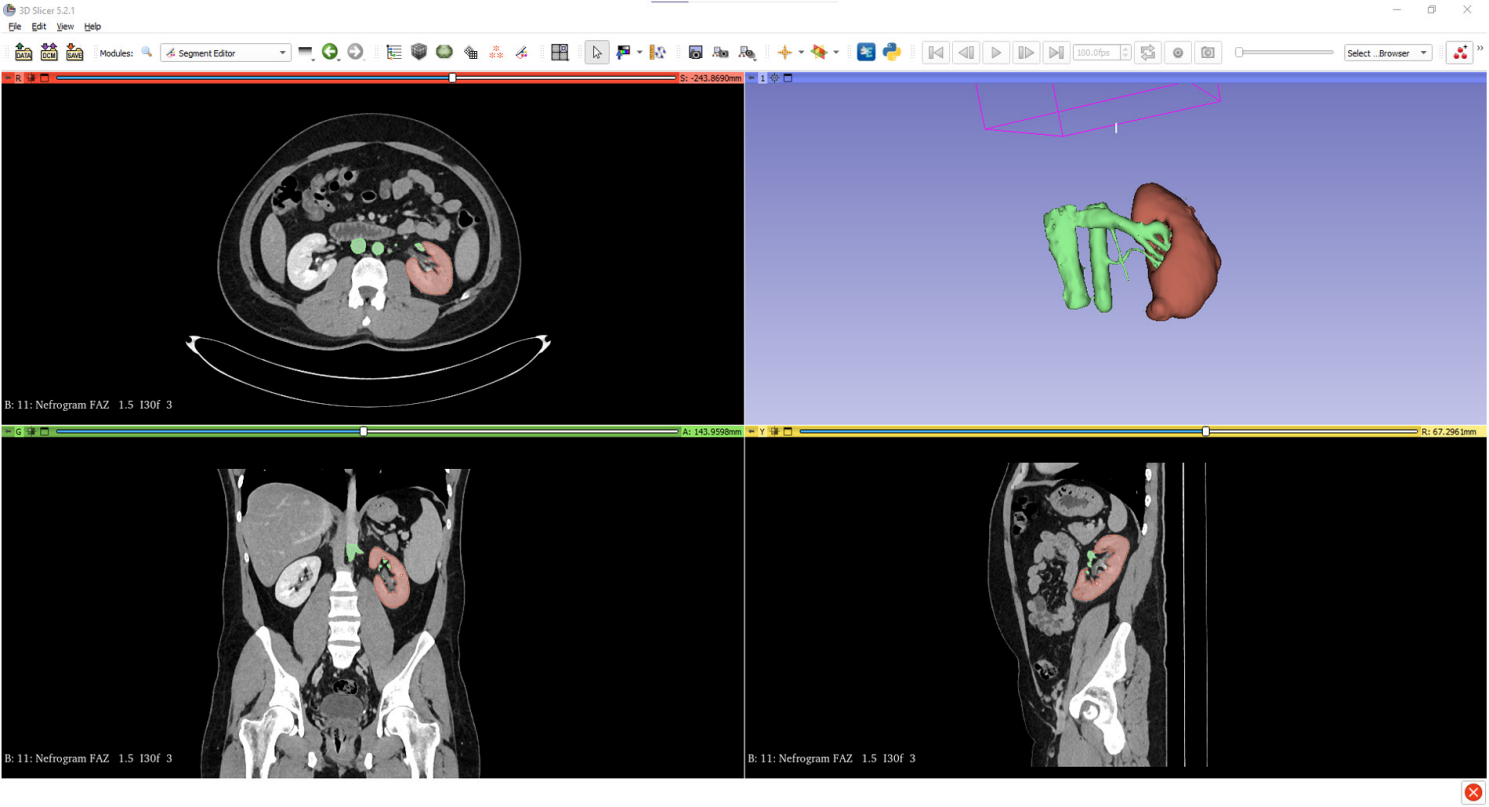
Segmentation images of the kidney tumor generated from CT scans using 3D Slicer software.

**Figure 2:**
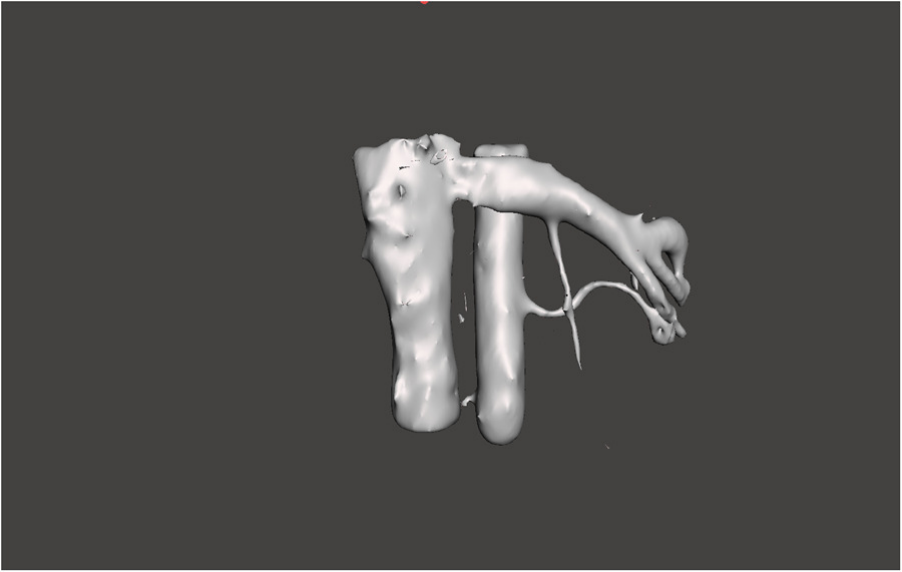
3D reconstruction of segmented renal artery and vein, post-processed using Meshmixer software.

**Figure 3:**
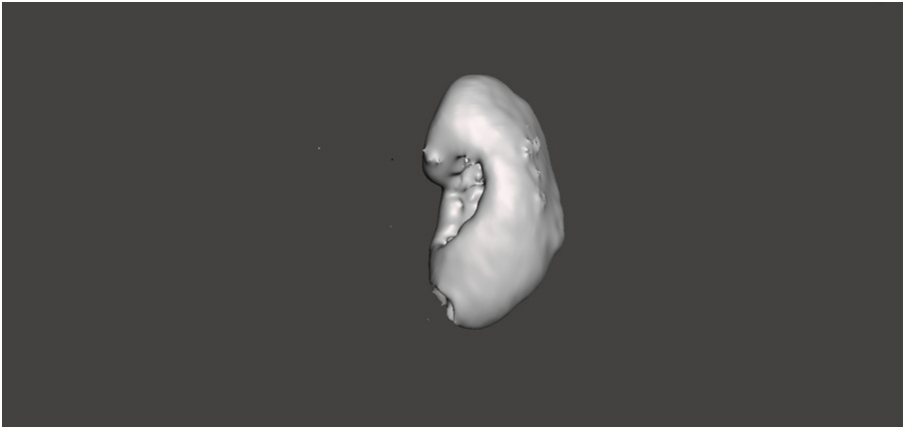
3D model of the renal parenchyma (excluding tumor), segmented and refined using Meshmixer software.

**Figure 4:**
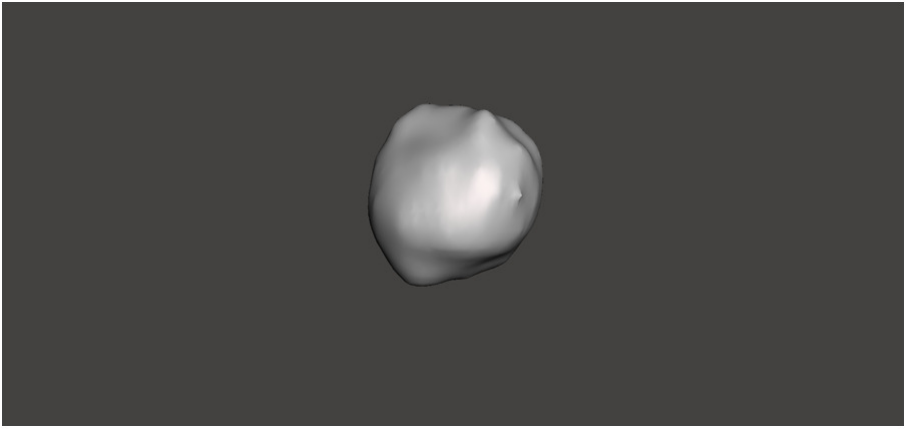
3D model of the renal tumor, segmented and refined using Meshmixer software.

**Figure 5:**
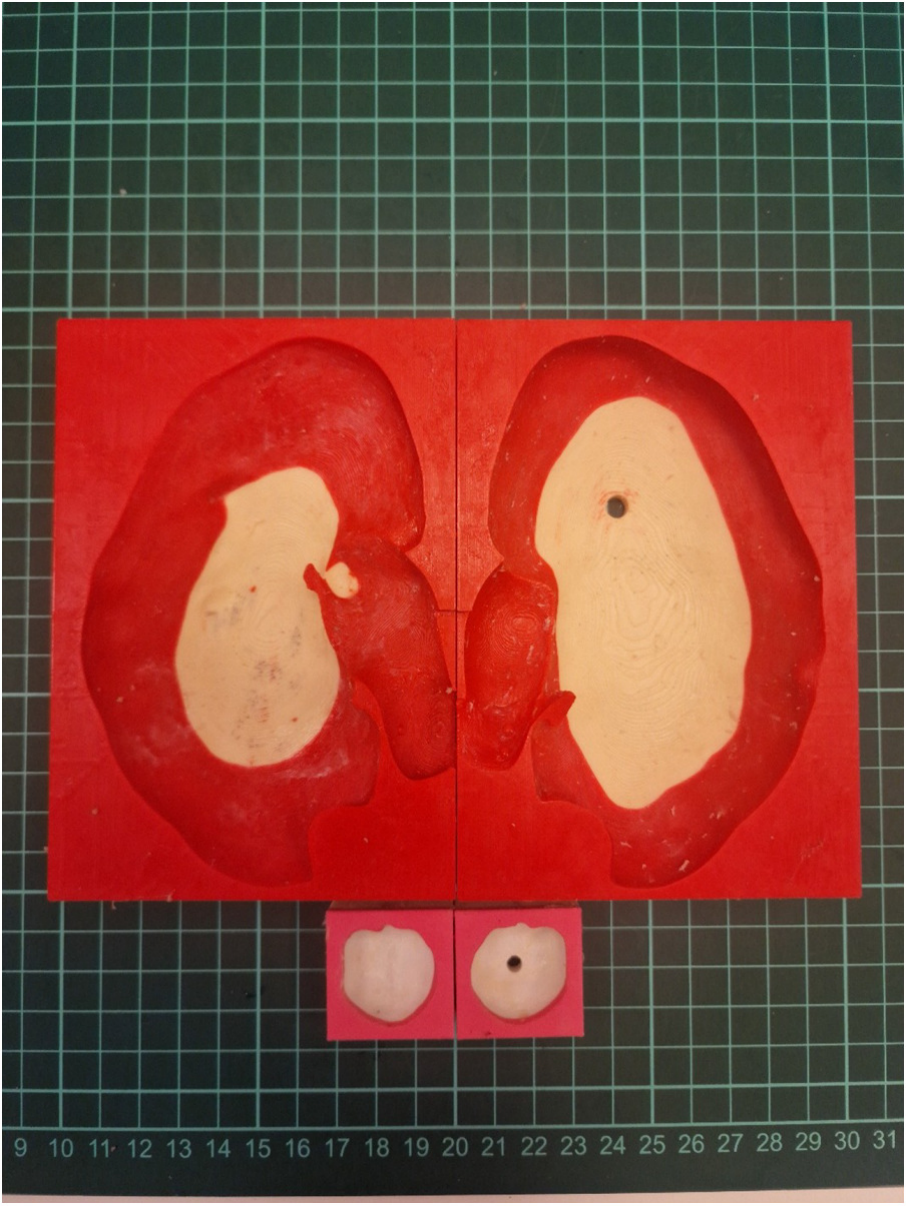
Negative molds and tumor components prepared for final model assembly.

**Figure 6:**
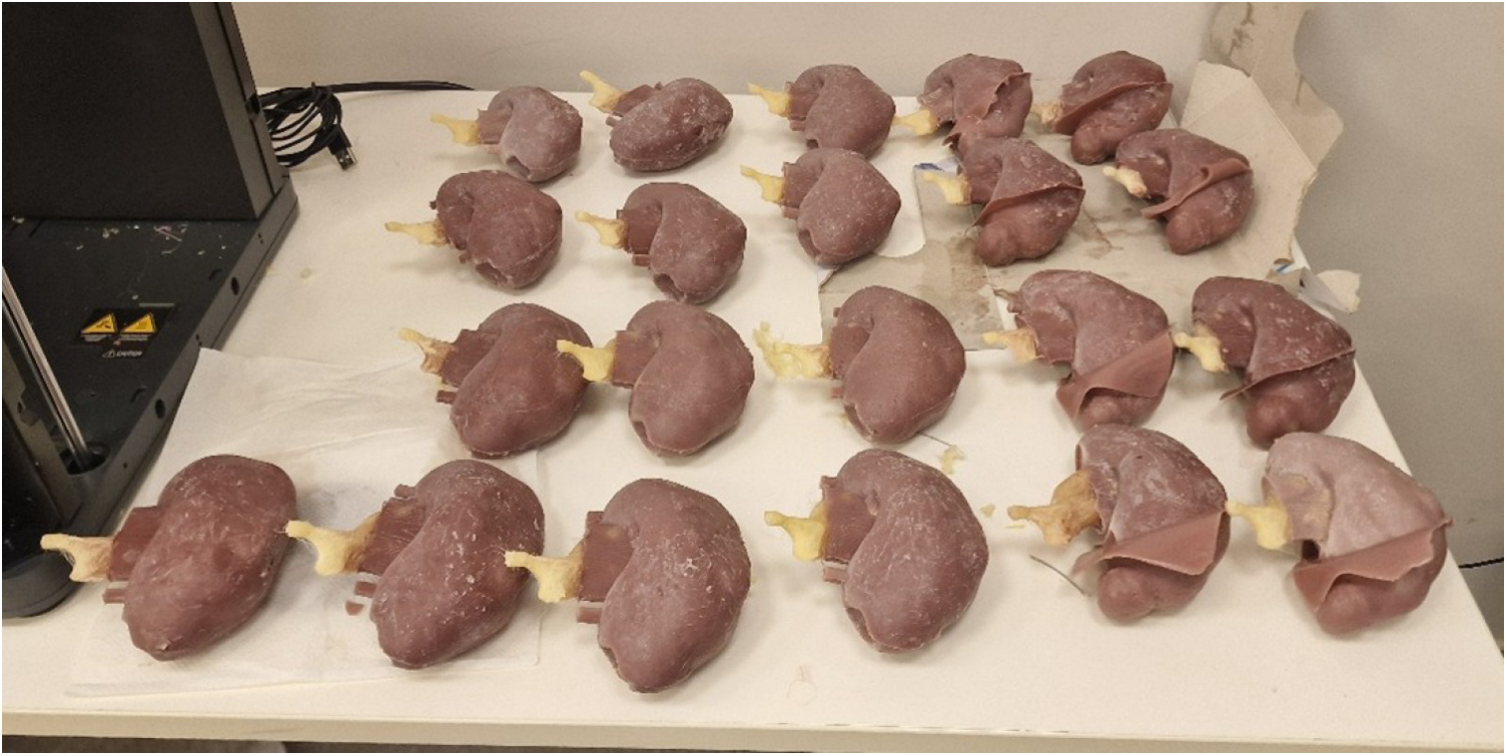
Finalized 3D-printed kidney models prepared for surgical simulation.

**Figure 7:**
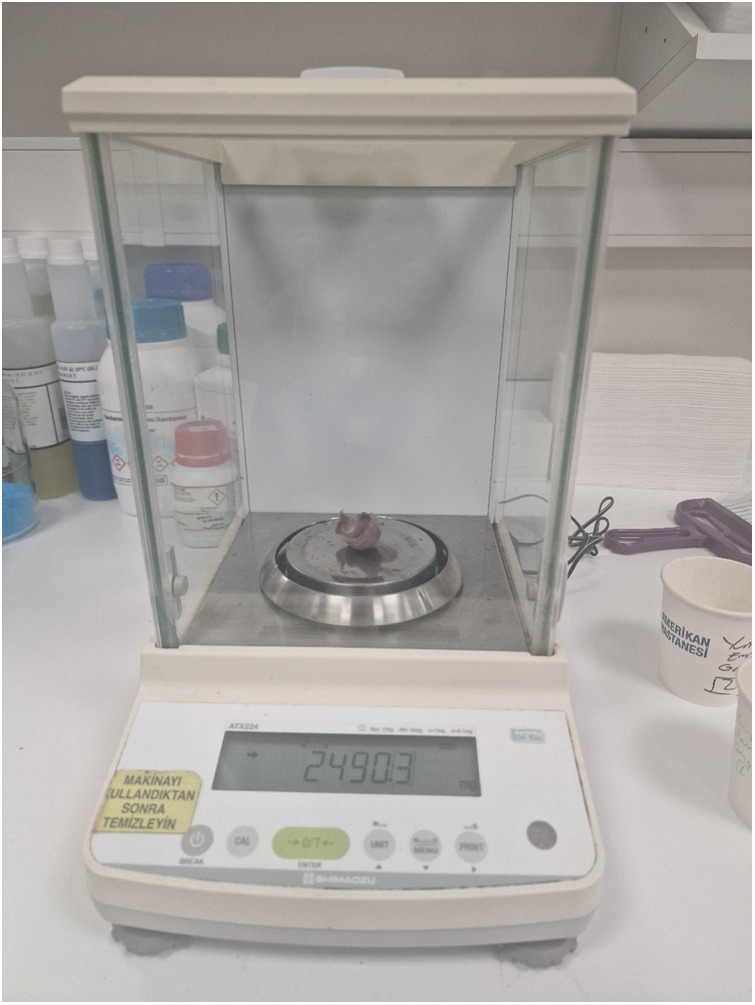
Measurement of resected healthy parenchyma using a precision laboratory scale.

In response, simulation technologies and three-dimensional (3D) printing have emerged as -innovative and ethical alternatives for acquiring surgical skills. Anatomical models created through 3D printing not only enhance surgical practice but also facilitate patient-specific surgical planning. While traditional training methods demand significant time and resources over the long term, simulation-based models and 3D printing technologies offer the potential to provide more efficient and cost-effective education. Furthermore, the integration of technologies such as virtual reality (VR) and augmented reality (AR) is expanding opportunities for hands-on training while creating a safer learning environment that minimizes errors.

Recent studies (3–7) have demonstrated that incorporating 3D printing technology into surgical education accelerates skill acquisition and improves understanding of complex anatomical structures. Individually customized models enable trainees to better appreciate patient-specific conditions and experience realistic surgical procedures. Additionally, training processes enhanced by personalized video feedback not only reduce surgical errors but also shorten the learning curve. By analyzing past performances to identify areas for improvement, this targeted approach further refines the training process.

This study aims to evaluate the impact of 3D printing technology on surgical education, examine the role of personalized video feedback in skill acquisition, and assess the integration of simulation-based training with traditional educational models across various surgical disciplines. The broader adoption of these innovative approaches may significantly enhance surgical training, ultimately contributing to the development of more competent and well-equipped surgeons.

## 2 Materials and methods

### 2.1 Study population and design

A total of 40 urology residents with no prior experience in robotic, open, or laparoscopic partial nephrectomy were invited from seven different centers to participate in this study.

Following successful completion of the European Basic Robotic Urological Skills (E-BRUS) theoretical course, developed by the European Association of Urology (EAU), participants proceeded to robotic simulation training in groups.

The simulation training incorporated the da Vinci Skills Simulator (dVSS; Intuitive Surgical, Sunnyvale, CA) with modules including Sea Spikes, Camera Targeting, and Needle Targeting (dV-Trainer, Mimic Technologies, Seattle, WA), as well as the Vertical Defect Suturing module (Simbionix, *currently, both Mimic Technologies and Simbionix have been acquired by Surgical Science.* An instructor guided each participant during the training sessions, continuing until proficiency was demonstrated in all simulator subsections. To ensure competency, participants were required to successfully complete each component of the assessment, including economy of motion and instrument collision evaluations.

After completing the structured training, participants were given additional time for unrestricted practice on the simulator, allowing them to practice freely without any time limitations.

Among the 40 participants, 20 residents were randomly selected to receive video-based feedback using recordings of their own surgical performances. The remaining 20 participants did not receive any feedback. Randomization was performed according to the order of arrival. One participant was unable to complete the final stage of the training and was therefore excluded from the study.

The primary parameters evaluated in this study included:

• Dissection time of the renal mass
• Repair time of the renal parenchyma defect (renorraphy)
• Total console time
• Amount of healthy renal parenchyma remaining following tumor excision from the removed specimen

Additionally, the duration of residency experience for each participant was recorded and analyzed.

The video feedback group included 11 junior and 9 senior residents, while the control group included 8 junior and 11 senior residents. Fisher's exact test indicated no statistically significant difference between the groups in baseline residency level (*p* = 0.527), confirming that they were comparable at baseline.

### 2.2 Material preparation

A renal mass with a low level of robotic surgical removal difficulty (low complexity) according to the R.E.N.A.L. Nephrometry Score (score 4a), was initially selected for this study (8). DICOM images of the renal mass were acquired and transferred to the 3D Slicer software. At this stage, segmentation, the process of defining the boundaries of the organs, was completed. For example, the mass and vascular structure in the lower pole were clearly delineated in the relevant sections.

Subsequently, the data were prepared for the negative printing technique using TinkerCad software. This technique involves injecting a prepared solution into a mold and then removing the solidified material from the mold, allowing for the mass production of identical models. Studies suggest that this method is the most effective approach currently available for producing tissue-like materials with realistic properties (9). Utilizing this injection molding method, a total of 80 identical models were produced. Molds were designed using TinkerCad and then fabricated with a Bambulab P1P model 3D printer.

For model construction, RTV (Room Temperature Vulcanizing) silicone was used with varying hardness levels. RTV type 10 Shore silicone, mixed with a silicone thinning agent, was used for the renal parenchyma, while RTV type 20 Shore silicone was used for the renal mass. A third mold facilitated the integration of the renal parenchyma and mass. During the curing process of the renal parenchyma molds, tissue representing the renal pelvis was incorporated. Polylactic Acid (PLA) filaments were used to create these molds, while PolyVinyl Alcohol (PVA) filaments were selected for the renal pelvis, offering the necessary flexibility.

All software utilized in this process was free and, where possible, open-source, significantly reducing costs by eliminating software licensing fees. Two models were produced for each participant, ensuring ample opportunity for hands-on practice.

Optional hilar structures, including the renal artery, renal vein, and ureter, could be added to the models based on 3D Slicer drawings. The inclusion of these structures incurred an additional cost of $0.50 to $2.00, depending on the materials used. During the production process, custom colors were applied to each structure using specialized paints, with an approximate paint cost of $0.056 per model.

The total production cost of a single kidney model was $2.06, with an additional electricity cost of $0.077, bringing the total cost to $2.14 per model. The initial cost of the molds was $4.63, and the PLA filament required for one-time mold production cost approximately $4.45. Over the course of the study, 80 models were produced, and participants had the option to request additional models if needed. Two participants utilized this option and received extra models during the study.

### 2.3 Operations and video feedback

The participants attended the first phase of the study at *RMK Academy of Interventional Medicine, Education, and Simulation (RMK AIMES*), where they observed a partial nephrectomy procedure performed by an experienced surgeon (AEC). Following this observation, they conducted a partial nephrectomy themselves using the Intuitive Da Vinci X model robotic system. The setup included one camera and robotic arms, operating within an abdomen model prepared using 3D printing technology.

All procedures were recorded using the Stryker InSITE Centre system. These recordings were later evaluated by a single experienced robotic surgeon (AEC) to ensure consistency in assessment. The primary focus of the evaluation was the tumor enucleation step during renal mass excision, where participants were encouraged to follow a true enucleation plane. In addition, technical deficiencies observed during renorrhaphy—such as improper needle angulation or failure to achieve the correct clip placement angle—were also identified. Personalized video feedback was created using these observations. Instructional annotations and verbal comments were added to each participant's surgical recording, emphasizing areas for improvement and reinforcing correct techniques. When participants returned to RMK AIMES within two weeks, the experimental group was shown their individualized feedback videos prior to the second attempt. The control group did not receive any feedback.

The video feedback provided to the experimental group included targeted instructional messages, such as:

• Guiding enucleation attempts when enucleation was required.
• Adjusting needle grip position, offering specific guidance on achieving the most accurate positioning.
• Confirming correct positioning, reinforcing positive behaviors.
• Providing optimal angles for ML sized (544,230) hem-o-lok clip (Teleflex Medical, Westmeath, Ireland) placement during renorrhaphy.

The primary outcome measures included:

• Dissection time
• Renorrhaphy time
• Total console time
• Amount of healthy parenchyma removed along with tumors

These parameters were compared between participants who received video feedback and those who did not.

To account for the varying stages of residency training among participants, the study initially evaluated the first operation parameters. This approach aimed to ensure that the pre-study training on 3D models placed all participants under comparable conditions before further evaluation.

During the study, a stopwatch was used to precisely measure the duration of each operational step. After each procedure, the tumoral formation was isolated from the excised mass, and the tissue representing the healthy parenchyma was weighed using the Shimadzu ATX224 precision balance (precision: 0.1 mg). This method enabled an accurate assessment of the effectiveness of nephron-sparing surgery performed by each participant.

### 2.4 Statistical analysis

SPSS version 28.0.0.0 (IBM, Chicago, USA) was used for statistical analysis. All numerical parameters were tested for normality using appropriate statistical methods. Comparisons between groups were performed using either the Mann–Whitney U test or the Student's t-test, depending on the distribution of the data. A *p*-value < 0.05 was considered statistically significant.

## 3 Results

Table 1 compares the first operations of junior and senior residents on 3D models after theoretical and simulation training. Since residency duration in Turkish urology programs is five years, participants were categorized as junior (<2.5 years of training) or senior (>2.5 years of training).

**Table 1:** Comparison of surgical performance parameters between junior and senior residents in their first operation on 3D kidney models after theoretical and simulation-based training.

Evaluated operative parameters	Junior residents	Senior residents	*P*-value
Dissection time (Op 1, sec) (SD)	365,74 (159,198)	390,10 (140,912)	0.336
Renorrhaphy time (Op 1, sec) (SD)	592,05 (191,314)	602,70 (179,675)	0.792
Total console time (Op 1, sec) (SD)	957,79 (263,492)	992,80 (252,226)	0.607
Amount of healthy parenchyma removed (Op1, mg) (SD)	3962,506 (757,9670)	3635,345 (898,2742)	0.158

The results analyzed in Table 1 are important because they show that simulation training with success condition after theoretical training eliminates the importance of the training time between the residents. These results showed us that having the success rates of the preparatory training to be done before the training to be done on 3D models can provide more accurate results. Here, there was no difference between the two groups in the first operations performed, which gives us a better starting point to examine the contribution of video feedback.

Table 2 compares the operative data of residents in the video feedback group and the control group.

**Table 2:** Comparison of surgical performance parameters between the video feedback group and the control group, including changes between the first and second operations on 3D kidney models.

Evaluated operative parameters	Video feedback group (*n* = 20)	Control group (*n* = 19)	*P* value
Dissection time (Op 1, sec) (SD)	394.85 (151.735)	360.74 (147.283)	0.481
Dissection time (Op 2, sec) (SD)	205.85 (121.005)	234.89 (75.562)	0.373
Dissection time improvement (sec) (SD)	189 (144.271)	125.84 (179.112)	0.232
Dissection time improvement (%) (SD)	46.63 (24.24)	23.62 (41.69)	0.043
Renorrhaphy time (Op 1, sec) (SD)	594.125 (180.166)	600.95 (190.924)	0.911
Renorrhaphy time (Op 2, sn) (SD)	371.05 (95.061)	416.37 (124.263)	0.207
Renorrhaphy time improvement (sec) (SD)	223.2 (171.81)	184.58 (149.162)	0.46
Renorrhaphy time improvement (sec) (SD)	33.6 (21.09)	27.9 (18.74)	0.379
Total console time (Op 1, sec) (SD)	989.1 (252.235)	961.68 (263.97)	0.742
Total console time (Op 2, sec) (SD)	576.9 (188.575)	651.26 (132.633)	0.165
Total console time improvement (sn) (SD)	412.2 (243.266)	310.42 (244.487)	0.201
Total console time improvement (%) (SD)	40.40 (16.59)	28.70 (19.84)	0.053
Amount of healthy parenchyma removed (Op 1, mg) (SD)	4,049.511 (1,145.5186)	3,531.458 (718.7944)	0.056
Amount of healthy parenchyma removed (Op 2, mg) (SD)	3,185 (901.3811)	3,124.095 (898.4014)	0.83
Improvement in the amount of healthy parenchyma removed (mg) (SD)	1,053.84 (985.44)	407.363 (959.1546)	0.048

In Table 2, the contribution of video feedback to training was analyzed. A statistically significant improvement was seen in the renal mass dissection time when examined in percentage of those who received a personalized video feedback during the surgical training process. This improvement did not reach statistical significance when analyzed in seconds, but when the pattern was carefully examined, improvement was also evident here. Again, during tumor removal, healthy parenchymal tissue, which was inadvertently removed, also showed a significant improvement after video feedback.

## 4 Discussion

The use of three-dimensional (3D) materials in medical education, particularly in anatomy training, has been extensively studied. Numerous studies have demonstrated that 3D models enhance anatomical understanding more effectively than traditional methods (3). Melnyk et al. further emphasized that 3D models provide more detailed information in a shorter time with reduced cognitive effort compared to CT scans (4). Beyond anatomy education, the application of 3D materials in surgical training is increasingly gaining attention, highlighting the importance of the properties of the 3D models used.

Conventional 3D printers primarily produce rigid materials, which, while adequate for anatomical education, may be less effective for surgical skill development. To address this limitation, our study employed silicone casting techniques using molds created via 3D printing. This method ensured that all training models were identical, providing a standardized platform for skill acquisition among participants.

The experience level of participants may also influence surgical performance. Since residency duration in Turkish urology programs is five years, participants were categorized as junior (<2.5 years of training) or senior (>2.5 years of training). Table 1 presents the procedural outcomes from the first attempt, showing no statistically significant differences between the two groups. This finding indicates that the e-learning module and proficiency-based simulation training provided at the beginning of the study effectively equalized the preparation level of participants, ensuring comparable performance regardless of residency duration.

Furthermore, the structured nature of the proficiency-based progression model used in this study may have contributed to the comparable baseline performance observed across participants. Unlike time-based simulation, where duration of exposure is fixed, our model required residents to demonstrate competency in each technical parameter before progressing (10). This ensured that all participants entered the surgical phase with a uniform minimum skill level. Such a competency-based approach is known to reduce inter-participant variability and create a more equitable training platform, particularly beneficial in studies comparing educational interventions such as video feedback (11–13).

Notably, the only critical error observed—tumor incision during dissection—occurred in a single participant. This outcome underscores the value of structured theoretical and simulation training as a reliable foundation for hands-on practice with 3D models.

As shown in Table 2, while the reduction in procedural time was not statistically significant, the observed trends remain clinically relevant. Analysis of operative duration and the amount of renal parenchyma excised demonstrated a significant reduction following video feedback. However, the lack of statistical significance may be due to the limited sample size. Per-participant analysis revealed statistically significant differences (*p* = 0.043), suggesting that video feedback contributes to improved surgical precision, particularly in enucleation attempts. In the initial procedures, many participants performed a combination of enucleation and resection (enucleoresection). Following video feedback, which emphasized adherence to true enucleation planes, a greater number of participants performed pure enucleation during the second procedures. This shift may account for the observed improvement in surgical precision. Our findings are consistent with Piramide et al., who reported that 3D models enhance enucleation efforts (14).

A comparison of renorrhaphy times indicated a trend toward improvement with video feedback, although the differences did not reach statistical significance. The impact of feedback was more pronounced in dissection times than in suturing times, likely reflecting the nature of surgical learning. Dissection involves learning new techniques and eliminating fundamental errors, whereas renorrhaphy primarily focuses on improving speed in an already familiar skill. This aligns with the principles of proficiency-based progression, a well-established approach in surgical education that has been shown to significantly reduce errors (12).

Our findings support this concept, demonstrating that video feedback is particularly beneficial for refining dissection techniques. Although improvements in surgical times were not always statistically significant, they highlight the potential benefits of integrating 3D models into training programs. This suggests that personalized training strategies may enhance skill acquisition more efficiently and cost-effectively.

Another key finding was that participants receiving video feedback removed significantly less healthy renal parenchyma than those without feedback (*p* = 0.048). This result correlates with the reduction in dissection time, suggesting that early feedback facilitates a more accurate understanding of the surgical plane. In complex procedures such as partial nephrectomy, unnecessary parenchymal removal often results from an inability to correctly identify dissection planes. Although limited to a single intervention, the observed improvements in dissection precision suggest that video feedback may facilitate a better understanding of the optimal surgical planes. Further studies are needed to confirm the durability and reproducibility of this effect.

Although limited research directly quantifies healthy renal tissue loss, prior studies have reported that using 3D models in partial nephrectomy reduces the frequency of collecting system entry, which aligns with our observations (14). Considering that robotic partial nephrectomy learning curves tend to plateau after approximately 150 cases (15), our findings suggest that case-specific training with 3D models may accelerate this learning curve.

The benefits of preoperative rehearsal using patient-specific models have been demonstrated in previous studies. For example, Ghazi et al. showed that patient-derived 3D models improved intraoperative outcomes in complex cases (16). This highlights the importance of model fidelity when designing patient-specific surgical simulations. When a 3D model is intended for preoperative rehearsal, meticulous attention to anatomical details is crucial to ensure an accurate and meaningful training experience. Additionally, suturable models capable of realistically simulating tissue handling during partial nephrectomy are highly beneficial.

Cost-effectiveness is another critical factor, as large-scale training programs require the production of multiple models. An ideal model should be rapidly producible, anatomically accurate, and affordable while closely mimicking real tissue characteristics.

To the best of our knowledge, this study presents the most cost-effective 3D-printed kidney model for surgical training (17–20), providing an accessible and scalable solution for enhancing surgical education and shortening the learning curve for complex procedures.

Our cost analysis revealed that our model offers a more economical alternative to commercially available options. By deriving models directly from DICOM data, we achieved high anatomical accuracy while maintaining cost efficiency. A notable advantage of our model is its rapid production timeline: within 12 h of a CT scan, the first training model can be fabricated, and within 24–48 h, sufficient models can be produced for an entire training cohort.

This capability enables case-specific surgical rehearsal, particularly for surgeons-in-training, and enhances patient education by allowing visualization of the planned procedure. Personalized training has been shown to improve skill acquisition and reduce surgical errors (21, 22).

Additionally, feedback-based training is a well-established method in surgical education (23, 24), and our findings suggest that its impact may be further amplified when integrated with advanced 3D models. Gallagher et al. demonstrated that proficiency-based progression yields significantly better results than traditional training methods (11, 25, 26). Our study supports this approach, indicating that the combination of 3D models and individualized feedback has the potential to enhance surgical education and optimize learning outcomes.

To the best of our knowledge, this is one of the largest studies evaluating the use of low-cost, anatomically accurate 3D-printed kidney models combined with video feedback in robotic partial nephrectomy training. Despite its promising results, one of the key limitations of the study is the inability to simulate intraoperative bleeding, which remains a challenge in current simulation technologies.

## Data Availability

The raw data supporting the conclusions of this article will be made available by the authors, without undue reservation.
